# Prenatal transplantation of mesenchymal stem cells to treat osteogenesis imperfecta

**DOI:** 10.3389/fphar.2014.00223

**Published:** 2014-10-09

**Authors:** Jerry K. Y. Chan, Cecilia Götherström

**Affiliations:** ^1^Experimental Fetal Medicine Group, Department of Obstetrics and Gynecology, Yong Loo Lin School of Medicine and National University of Singapore, Singapore, Singapore; ^2^Department of Reproductive Medicine, KK Women’s and Children’s Hospital, Singapore, Singapore; ^3^Cancer and Stem Cell Biology, Duke-National University of Singapore Graduate Medical School, Singapore, Singapore; ^4^Division for Obstetrics and Gynecology, Department of Clinical Science Intervention and Technology, Karolinska Institutet, Stockholm, Sweden; ^5^Center for Hematology and Regenerative Medicine, Karolinska Institutet, Stockholm, Sweden

**Keywords:** prenatal transplantation, *in utero* transplantation, intrauterine transplantation mesenchymal stem cells, fetal stem cells, osteogenesis imperfecta

## Abstract

Osteogenesis imperfecta (OI) can be a severe disorder that can be diagnosed before birth. Transplantation of mesenchymal stem cells (MSC) has the potential to improve the bone structure, growth, and fracture healing. In this review, we give an introduction to OI and MSC, and the basis for pre- and postnatal transplantation in OI. We also summarize the two patients with OI who have received pre- and postnatal transplantation of MSC. The findings suggest that prenatal transplantation of allogeneic MSC in OI is safe. The cell therapy is of likely clinical benefit with improved linear growth, mobility, and reduced fracture incidence. Unfortunately, the effect is transient. For this reason, postnatal booster infusions using same-donor MSC have been performed with clinical benefit, and without any adverse events. So far there is limited experience in this specific field and proper studies are required to accurately conclude on clinical benefits of MSC transplantation to treat OI.

## OSTEOGENESIS IMPERFECTA

Osteogenesis imperfecta (OI), or brittle bone disease, is a group of genetic disorders caused mainly by defects in collagen synthesis (Forlino and Marini, 2000). The majority of OI cases are caused by some 1500 different dominant mutations in the COL1A1 or COL1A2 gene, resulting in abnormal assembly of the protein (Canty and Kadler, 2005). More recently, recessive forms for OI has been described, where defects in proteins involved in post-translational modifications or transport leading to perturbations of the collagen 3-hydroxylation complex (Barnes et al., 2010), and collagen chaperone pathways (Alanay et al., 2010; Christiansen et al., 2010). OI presents in a clinically heterogenous manner, ranging from the mild type I, to the progressively deforming type III and the perinatally lethal type II according to the original Sillence classification (Sillence et al., 1979). An evolving understanding of the genetics has now made it possible to consider refining and adding to the original Sillence classification (Forlino et al., 2011).

Currently, the goal of clinical management is to optimize the patient’s gross motor abilities and to achieve a level of independence during childhood life. This is largely accomplished empirically through physical rehabilitation and life-long orthopedic interventions in correcting bony deformities of the long bones and vertebra (Laron and Pandya, 2013). Pharmacological intervention is underpinned by the use of bisphosphonates in an effort to reduce bone resorption and increase bone mineralization (Rauch and Glorieux, 2006). While mineralization has been shown to improve with bisphosphonate treatment, a recent meta-analysis of randomized trials failed to demonstrate consistent benefits in fracture rates, reduction of pain, or functional mobility (Dwan et al., 2014). Moreover, there is a growing concern on the role of bisphosphonates in impairing bone remodeling in these children (Marini, 2009). Growth hormones have also been evaluated, and have shown encouraging benefits in the increase in linear growth (Antoniazzi et al., 1996), and are being considered for treatment in combination with bisphosphonates (Antoniazzi et al., 2010).

Due to the lack of effectiveness in current modalities of treatment, which does not address the underlying molecular defect, alternative approaches are currently being explored. Some of these experimental treatments include allogeneic cell transplantation (Horwitz et al., 1999, 2001, 2002; Otsuru et al., 2012). The genetic defect may be corrected through homologous recombination of the patient’s stem cells (Chamberlain et al., 2004), or through the degradation of abnormal COL1A1/2 transcripts (Millington-Ward et al., 1997, 2002). In this paper, we will focus on the use of allogeneic mesenchymal stem cells (MSC) in pre- and postnatal treatment of OI.

## MESENCHYMAL STEM CELLS AS DONOR CELLS IN OI

Mesenchymal stem cells are stromal cells that have been originally identified from the adherent portion of bone marrow (Friedenstein et al., 1966, 1968). They grow as spindle shaped cells displaying colony-forming capacity in low density cultures and are non-hematopoietic and non-endothelial. MSC can be propagated through multiple passages in cell culture and differentiate down the standard osteogenic, adipogenic and chondrogenic lineages under permissive conditions (Pittenger et al., 1999). MSC do not express human leukocyte antigen (HLA) class II antigens, and are generally considered to be non-immunogenic in nature and possess immune-modulatory properties (Le Blanc, 2003). Although attempts at standardizing the nomenclature of what constitute a MSC has been proposed by the International Society of Cellular Therapy (Dominici et al., 2006), evidence of bona fide stem cell properties had only been demonstrated in CD146 positive perivascular cells in human bone marrow with *in vivo* self-renewal properties (Sacchetti et al., 2007). Over the past couple of decades, the isolation of MSC-like cells has been reported from multiple organs and tissues (da Silva Meirelles et al., 2006), which may originate from NG2 (neuron-glial antigen 2) and CD146 positive pericytes (Crisan et al., 2008), although non-bone marrow-derived MSC have yet to be validated for stemness.

As MSC are readily isolated and expanded, are non-immunogenic and have multilineage differentiation capacity, they have been studied and indeed trialed clinically in a diverse range of clinical scenarios ranging from the treatment of graft versus host disease (Le Blanc et al., 2008), autoimmune diseases such as Crohn’s disease (Uccelli et al., 2008; Uccelli and Prockop, 2010), cardiovascular diseases like acute myocardial infarction and stroke and orthopedic applications including bone and cartilage repair (Zhang et al., 2012). However, MSC have limited ability to be expanded under standard conditions especially with increasing age (Siegel et al., 2013; Choudhery et al., 2014), rapidly senescing in culture and with restrictions in multilineage differentiation capacity (Digirolamo et al., 1999; Muraglia et al., 2000). In addition, MSC yield, growth kinetics and differentiation capabilities vary significantly between individuals, restricting their clinical utility (Javazon et al., 2004).

Another class of MSC has been characterized in fetal and perinatal life, initially from first trimester tissues (Campagnoli et al., 2001; Gotherstrom et al., 2003; Chan et al., 2008), and have now been found in multiple tissue types including the umbilical cord, placenta, and amniotic fluid (O’Donoghue and Chan, 2006; De Coppi et al., 2007). These primitive MSC types have been found in higher frequencies with a higher colony-forming capacity, having longer telomeres and a higher prolif-erative potential (Chan et al., 2005; Guillot et al., 2007), and differentiate more readily into bone and muscle (Chan et al., 2006, 2007; De Coppi et al., 2007; Zhang et al., 2009), and non-mesenchymal lineages such as neural and hepatic cells (De Coppi et al., 2007; Kennea et al., 2009). Like their adult counterparts, human fetal MSC (hfMSC) are also non-immunogenic, and have the ability to modulate immune responses (Gotherstrom et al., 2003, 2004; Gotherstrom, 2007; Di Trapani et al., 2014). Given the ability of hfMSC to be expanded several fold more efficiently than adult MSC, their enhanced colony-forming capacity and increased bone differentiation capacity, they may be the ideal cell type for treatment of OI.

The rationale for the use of allogeneic MSC for the treatment of OI is underpinned by the ability of MSC to home to bone (Pereira et al., 1995; Guillot et al., 2008) and indeed to regions of active remodeling as found in fracture sites and in patients with OI. MSC secretes both paracrine growth factors and normal type I collagen conducive for generating normal bone tissues and thus ameliorating the bone fragility phenotype in OI.

## PRENATAL TRANSPLANTATION

Skeletal dysplasia occurs in fetal life, which can be diagnosed readily through mid-trimester fetal anomaly scans (Schramm et al., 2009; Barkova et al., 2014). Affected fetuses may present with shortened long bones and the occurrence of multiple fractures, alerting the clinician toward a diagnosis of OI. Definitive prenatal genetic diagnosis may be achieved through standard amniocen-tesis or fetal blood sampling, both of which are established fetal medicine procedures largely available in most developed countries. This opens up the possibility of offering prenatal treatment.

In the context of OI, the most convincing argument for prenatal transplantation would be to ameliorate the disease process at a time of rapid skeletal development where spontaneous fractures are occurring. Other arguments in favor of a prenatal approach includes (i) the relatively smaller cell doses required due to the size of the fetus, (ii) the shunting of the intravenously delivered cells to the arterial circulation through the patent foramen ovale in fetal life rather than being trapped in the lungs in postnatal life, and (iii) possibly the lower risks of immune rejection in the developing immune system of the recipient (Lee et al., 2009; Mattar et al., 2012).

## PRENATAL TRANSPLANTATION IN ANIMAL MODELS OF OSTEOGENESIS IMPERFECTA

Experimental evidence of the efficacy of prenatal MSC transplantation was provided by Guillot and colleagues, who tested the ability of first trimester fetal blood-derived MSC to ameliorate OI in the *oim* mouse, a naturally occurring recessive mouse model approximating human type III OI, with progressive deformities and skeletal fractures (Chipman et al., 1993). In this model, 10^6^ culture expanded hfMSC were injected intraperitoneally at E14 gestation (a high dose of around 10^9^/kg fetal weight) and allowed to litter naturally in this xenogeneic transplantation model in a fully immune-competent recipient (Guillot et al., 2008). Donor cells engrafted in a wide range of tissues such as the skin, heart, lung, brain, and thymus, but were found in greater quantities in skeletal tissues where up to 5% of cells were of donor origin. Human donor cells expressing the bone marker osteopontin tended to cluster around areas of active bone formation and at fracture sites. Transplanted mice demonstrated improved bone strength, length, and cortical thickness, with a two-third reduction in fractures (Guillot et al., 2008).

More recently, Panaroni et al. (2009) investigated the ability of prenatal allogeneic bone marrow transplantation in the BrtlIV mouse, a dominant model of OI more reflective of human type II OI. Here, 5 × 10^6^ unmanipulated bone marrow from adult donors was transplanted intraperitoneally to E13.5-E14.5 fetuses where wild type females were mated with heterozygous BrtlIV males, which should produce affected fetuses in half the litter. The transplantation resulted in rescue of perinatal lethality, as transplanted mice had a higher proportion of surviving BrtlIV offspring. At 2 months of age, only 64% of transplanted mice were chimeric for donor cells in multiple hematopoietic tissues including bone marrow where donor cells accounted for 1-2% of all cells, and produced up to 20% of the bone collagen. Donor cells were found in clusters in long bones, with accompanying improvement in bone mineral density and cortical thickness in treated compared to untreated BrtlIV mice. Thus, these two models provide evidence supporting a prenatal approach to treat of OI, leading to higher engraftment rates, amelioration of disease phenotype and rescue of perinatal lethality. In addition, data from the BrtlIV mouse study suggests that significant amount of normal collagen can be deposited by a relatively small population of chimeric donor cells. This would explain the marked improvements in mineralization and growth seen in the clinical transplantation cohort where engraftment levels are generally around 1%.

## CLINICAL EXPERIENCE OF POSTNATAL CELL TRANSPLANTATION IN OSTEOGENESIS IMPERFECTA

The first clinical proof of principle of an allogeneic stem cell transplantation approach came from Horwitz and colleagues where children affected with type III OI underwent transplantation with unmanipulated bone marrow from HLA-identical or single-antigen-mismatched siblings after ablative conditioning therapy. The treated children exhibited increased linear growth velocities and reduced fracture frequencies in spite of the low frequency (<2%) of donor osteoblast engraftment in the bone (Horwitz et al., 1999, 2001). The same group carried out further clinical studies with MSC isolated from the same bone marrow donors. This study included six children at age 2-4 years that received two infusions of 1 - 5 × 10^6^/kg HLA-matched gene marked adult MSC. It resulted in MSC engraftment and an increase in linear growth velocities (Horwitz et al., 2002). Thus, it was established here that allogeneic MSC infusion is safe in the context of OI, with donor cells engrafting in bone and results in an increase in mineralization and growth velocity, albeit only for a limited time. Although the same group later showed that the origin of donor blasts might be from the non-adherent fraction of bone marrow, i.e., the hematopoietic stem cells, rather than the adherent fraction from where MSC are generally isolated (Otsuru et al., 2012). Not withstanding that, MSC can contribute to bone growth and mineralization, possibly through secreted growth factors (Otsuru et al., 2012). Moreover, it would be inconceivable to put a child through a bone marrow transplantation procedure with all its attendant morbidity and mortality when the use of non-matched MSC would obviate the need for conditioning therapy, and have shown an excellent safety profile through thousands of clinical infusions (Aguilar et al., 2007; Zhang et al., 2012).

## CLINICAL EXPERIENCE OF PRENATAL MSC TRANSPLANTATION IN OSTEOGENESIS IMPERFECTA

Promising results of tissue repair in animal studies have led to numerous clinical studies using MSC to treat severe disorders and several reports indicate a role for MSC therapy in the treatment of OI both pre- and postnatally. As described above, Horwitz et al. (2002) performed the first study using HLA-matched MSC to treat OI postnatally. Results were promising showing low toxicity, engrafted donor cells and accelerated growth. Encouraged by this study, prenatal transplantation using hfMSC has since then been reported in two cases of OI.

The first case presented at gestational week 15 and was later diagnosed as OI type III, which postnatally was confirmed with genetic analysis (COL1A2 c.3008G>A; p.Gly1003Asp; Gly913Asp in the triple helical domain; Le Blanc et al., 2005; Gotherstrom et al., 2014). At week 24, all limbs were -5 SD and angulated, with femoral fractures noted. The baby was infused with 6.5 × 10^6^/kg HLA-unmatched hfMSC at gestational week 31. At 4 months of age, bisphosphonate treatment was initiated due to presence of vertebral compression fractures. Until 8 years of age, she was doing acceptably well with little more than one fracture and one compression fracture per year (5 femoral, 2 clavicular, 1 shoulder and 1 skull fracture and 11 vertebral compression fractures). Remarkably she continued growing and followed her own height and weight growth curve at -5 SD until the age of 6, when it had deteriorated to -6.5 SD at the age of 8 years. Due to the increased fracture rate and declined growth, the patient was transplanted with 2.8 × 10^6^/kg same-donor cells at the age of 8 years. The subsequent 2 years after the re-transplantation the patient did not suffer from any new fractures and the linear growth and mobility improved (she was able to walk 1000 m without difficulties, started dance classes, increased her participation in gymnastics at school). Donor osteoblastic cells were detected in the bone, but not in any other tissues, at 9 months and 9 years of age. The level of engraftment was varying, between 0.003 and 16.6%. Only one other patient is currently known to have an identical COL1A2 mutation and presented with a very severe phenotype of OI. This patient did not receive MSC therapy and succumbed at 5 months of age despite postnatal bisphosphonate therapy.

The second case was a baby with OI type IV who presented with short long bones (<5th centile) and multiple fresh and healing fractures at 26 weeks of gestation (Gotherstrom et al., 2014). The baby was transplanted with 30 × 10^6^/kg HLA-unmatched hfMSC at 31 weeks of gestation, and did not suffer any new fractures for the remainder of the pregnancy or during infancy. The patient’s family had a history of short stature and multiple fractures and genotyping of the patient and family members identified an autosomal dominant mode of inheritance (c.659G>A; p.Gly220Asp, Gly130Asp in the triple helical domain). No donor cells were detected in umbilical cord blood, umbilical cord, and placenta. There have been no opportunities to obtain bone samples for analysis in this case. Bisphosphonate therapy was initiated from 1 month of age due to poor mineralization. The patient followed her own growth curve until 12 months of age (just below the 3rd centile), where longitudinal length plateaued. A postnatal infusion of 10 × 10^6^/kg MSC from the same donor was performed at 19 months of age, resulting in resumption of her growth trajectory and she continued to grow just below the 3rd centile. She started to walk shortly after the transplantation.

Similarly as described in the study by Horwitz and colleagues, the above described pre- and postnatal transplantations report a transient clinical effect after hfMSC infusion (Horwitz et al., 2002; Le Blanc et al., 2005; Gotherstrom et al., 2014), and several repeated transplantation might be required during the patients lifetime, especially in childhood. Nevertheless, intravenous infusion of same-donor hfMSC pre- and postnatally appears safe. The reported follow-up period is 3-10 years after prenatal transplantation and 2-2.5 years after postnatal transplantation. There were no signs of any adverse early or late reactions. There was no alloreactivity of the patient’s lymphocytes detected toward the donor hfMSC. Before the re-transplantations, analysis showed the absence of antibodies directed toward HLA class I and II, IgG and IgM, or fetal bovine serum (FBS).

The cell dose is a critical parameter in cell transplantation since it may relate to efficacy but a high cell dose may cause toxicity. In the reported hfMSC transplantations, the cell dose varied from 5 × 10^6^ to 30 × 10^6^/kg at prenatal transplantation and from 2.8 × 10^6^ to 10 × 10^6^/kg at postnatal transplantation. All doses were well tolerated, however, it is unclear from this data on two patients or from data on adult MSC transplantation for other disorders if a high cell dose is more efficacious. This remains to be investigated.

## SUMMARY

The two cases described here suggest the safety and feasibility of prenatal transplantation using HLA-mismatched hfMSC. Furthermore, it suggests a potential benefit to children with OI. However, the benefit from a single transplant before birth was transient and subsequent boosters with same-donor cells were performed with good effect. This is in line with the results from the study on postnatal MSC therapy in OI by Horwitz et al. (2002). The summarized data highlights the need to modify the pre- and postnatal transplant strategies, and possibly the cells, in order to improve MSC homing and engraftment for better long-term outcomes.

To accurately evaluate if pre- and postnatal MSC therapy in OI is an effective treatment, coordinated studies and joint efforts are necessary. Development of common programs, registries and guidelines (cell source, isolation and expansion and release criteria, patient inclusion criteria, transplantation strategies, follow-up measures, etc.), as well as coherence between investigators within the field is of significant importance. Considering the complexity of the disease and procedure, we see this as the only realistic way to proceed if we are to accurately evaluate the potential of MSC therapy in OI.

The summarized cases demonstrate that prenatal transplantation of allogeneic hfMSC and postnatal boosters using same-donor cells in OI is safe. The MSC infusions appear to give clinical benefit, although transiently. However, so far we have limited experience and further studies are required.

## AUTHOR CONTRIBUTIONS

Jerry K. Y. Chan and Cecilia Götherström drafted, wrote, and revised the manuscript. Both authors approved the final version of the manuscript.

### Conflict of Interest Statement

The authors declare that the research was conducted in the absence of any commercial or financial relationships that could be construed as a potential conflict of interest.
